# Tear film layers and meibomian gland assessment in patients with type 1 diabetes mellitus using a noninvasive ocular surface analyzer: a cross-sectional case–control study

**DOI:** 10.1007/s00417-022-05934-w

**Published:** 2022-12-13

**Authors:** María-Carmen Silva-Viguera, Alicia Pérez-Barea, María-José Bautista-Llamas

**Affiliations:** 1 Department of Physics of Condensed Matter, Optics Area, Physics Faculty, University of Seville, Reina Mercedes St, Seville, Spain; 2 Vision Research Group (CIVIUS), University of Seville, Seville, Spain

**Keywords:** Dry eye disease, Meibomian glands dysfunction, Ocular surface, Tear film, Type 1 diabetes mellitus

## Abstract

**Purpose:**

To assess the tear film layers and Meibomian glands by a noninvasive ocular surface analyzer in patients with and without type 1 diabetes mellitus (T1DM).

**Methods:**

Eighty-eight participants were enrolled in this study: 44 patients with T1DM without diabetic retinopathy, and 44 patients as a control group, between 18 and 49 years old. Limbal and bulbar redness classification, lipid layer thickness (LLT), tear meniscus height (TMH), first and mean noninvasive tear break-up time (FNIBUT and MNIBUT, respectively), and Meibomian glands loss (MGL) were assessment through the ICP Ocular Surface Analyzer (OSA). Schirmer’s I test (SIT), the fluorescein tear break-up time test (TFBUT), OSDI and SPEED questionnaires, and percentage of glycosylated hemoglobin (HbA1c) were also tested.

**Results:**

The T1DM group showed higher limbal and bulbar redness (*p* = 0.010) and lower LLT (*p* < 0.001), TMH (*p* < 0.001), FNIBUT (*p* < 0.001), MNIBUT (*p* < 0.001), SIT (*p* = 0.001), and TFBUT (*p* < 0.001) than the control group. A higher percentage of MGL was found in the T1DM group in the upper (*p* = 0.097) and lower (*p* < 0.001) eyelids. No significant differences were found in dry eye symptoms across the OSDI and SPEED questionnaires between the two groups.

**Conclusion:**

Patients with T1DM without signs of retinopathy showed involvement of the mucoaqueous and lipid layers of the tear film, as well as a higher percentage of MGL, using a noninvasive analyzer. Dry eye disease in people with T1DM cannot be ruled out by anamnesis and subjective symptom questionnaires alone; therefore, these patients should undergo regular anterior pole examinations.

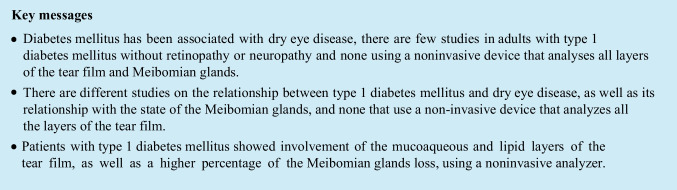

## Introduction

Diabetes mellitus (DM) is a systemic and chronic degenerative disease characterized by chronic hyperglycemia due to deficiency in the production or action of insulin, affecting the metabolism of carbohydrates, proteins, and fats. In type 1 diabetes mellitus (T1DM), the β cells of the pancreas are destroyed, which leads to an absolute insufficiency of insulin in the blood [1]. It can affect practically all ocular structures, with corneal dysfunction being the main complication in the anterior segment, with the appearance of abnormal sensitivity, delayed corneal re-epithelialization, diabetic keratopathy, progressive decrease in density and/or alteration of the corneal nerve, and opacity of the lens at a younger age [2].

We can also find alterations of the tear film, the function of which is to lubricate and maintain a smooth and refractive surface for optimal visual performance. Dry eye disease (DED) is defined as a disorder of the tear film and the ocular surface caused by insufficient tears or excessive evaporation of the same, which produces damage to the interpalpebral ocular surface and symptoms of discomfort [3].

The stability of the tear film can be assessed with different tests, such as the Schirmer test, the invasive tear break-up time, noninvasive tear break-up time (NIBUT), lipid layer thickness (LLT), or the tear meniscus height (TMH). The measurement of these parameters is important to diagnose DED. Normally, an observer takes measurements using the slit lamp and the use of fluorescein to assess the tear break-up time (TFBUT), which implies that the measurement is not entirely reliable. These tests evaluate different aspects of the tear, such as its production or quality, to classify DED into tear deficiency or excessive evaporation. In the latter, the Meibomian glands (MG) play an important role; Meibomian glands dysfunction (MGD) is commonly characterized by terminal duct obstruction and/or qualitative/quantitative changes in glandular secretion and is the leading cause of evaporative DED [2].

Eyes with an abnormally low tear film break-up time and the presence of subjective symptoms are considered to have DED; however, the Dry Eye Workshop II (DEWS II) of the Tear Film and Ocular Surface Society (TFOS) has recently published dry eye definition as follows: “Dry eye is a multifactorial disease of the ocular surface characterized by a loss of homeostasis of the tear film, and accompanied by ocular symptoms, in which tear film instability and hyperosmolarity, ocular surface inflammation and damage, and neurosensory abnormalities play etiological roles” [4]. Stapleton F et al. in a meta-analysis concluded that the prevalence of dry eye can range from 5 to 50% depending on age, sex, race, or geographic region analyzed [5]. The study by Millán et al., on the 11-year incidence of dry eye and risk factors in a cohort of 209 adults in Spain, obtained an incidence of 25.4% and 31.6% in signs and symptoms of dry eye, respectively, significantly associated with age (*p* < 0.05). In addition, they also concluded that some factors may increase the risk of dry eye signs and others of dry eye symptoms [6].

Therefore, it is necessary to make evaluations of the tear film as objective as possible. Newer diagnostic tests, such as tear film osmolality, refractive meniscometry for measurement of TMH, infrared meibography, interferometry to analyze the lipid layer of the tear film, the stability of the tear film (video keratography), and ocular surface thermography, are some of the instruments used to objectify these examinations.

There are different studies on the relationship between diabetes and DED. Although most of these studies have been carried out in patients with type 2 diabetes mellitus (T2DM) [7–9] or in patients with T1DM in childhood [10, 11], none of them presents objective data of patients with T1DM as well as their relationship with the state of the MG.

Although DM has been associated with DED, there are few studies in adults with T1DM without retinopathy or neuropathy and none using a noninvasive device that analyzes all layers of the tear film and Meibomian glands. The diagnosis of DED aims not only to detect this pathology to treat it but also to reduce the factors that lead to serious corneal complications that can compromise vision, such as corneal inflammation, corneal abrasions, or ulcers [3].

The aim of this research was to assess the tear film layers and meibomian glands by a noninvasive ocular surface analyzer in patients with and without T1DM.

## Material and methods

### Study design and ethics

A cross-sectional, case–control study was carried out in the Optometry Facilities of the Faculty of Pharmacy of the University of Seville (Seville, Spain) between January and May 2022. The study was conducted according to the ethical principles for medical research set out in the Declaration of Helsinki and was approved by the Ethical Committee of Andalusia (code 1474-N-19).

### Selection of participants

For the selection of participants with T1DM, a proposal for participation in the study was sent by email to the association of diabetics of Seville (ANADIS). In addition, an age- and sex-matched control group was selected among volunteers of the university community of the Faculty of Pharmacy of Seville. All participants signed an informed consent form after an explanation of the nature and consequences of the study.

The inclusion criteria were as follows: (1) age between 18 and 50 years; (2) absence of systemic or ocular disease; (3) absence of pharmacological prescription except for T1DM; and (4) absence of a previous history of ocular surgery.

The exclusion criteria were as follows: (1) ocular infection or inflammation; (2) taking any ophthalmic or systemic medications with tear film or ocular surface effects; (3) pregnant or breastfeeding patients; (4) a recent history of contact lens wearing; and (5) use of any ocular lubricant 1 week before.

Subjects with T1DM were controlling their DM with insulin injections, had to be diagnosed by a diabetes physician specialist with the disease at least 3 years earlier, and had no signs of retinopathy on fundus imaging according to the Early Treatment of Diabetic Retinopathy Study (ETDRS) [12]. Controls should have a percentage of capillary glycosylated hemoglobin (HbA1c) of no more than 5.6%.

### Material and measurements

The symptomatology of dry eye was evaluated using two subjective questionnaires: the Ocular Surface Disease Index (OSDI) [13] and the Standard Patient Evaluation of Eye Dryness (SPEED) test [14]. A nonmydriatic retinography (CSO nonmydriatic fundus camera Cobra HD, Italy) examination was performed to rule out the presence of signs of diabetic retinopathy. HbA1c was also tested in all subjects using the Cobas b-101 analyzer (Roche Diagnostic) to determine glycemic control. HbA1c defines the average blood glucose level of the previous 2–3 months, and in patients with DM, it reflects the success of diabetes management [15].

The noninvasive analysis of the tear film was performed through the Integrated Clinical Platform (ICP) Ocular Surface Analyzer (OSA) from SBM System® (Orbassano, Torino, Italy) [16–18]. OSA provides a complete noninvasive assessment of the ocular surface by combining several tests for the diagnosis of DED. This device was placed in the tonometer hall of the slit lamp. The measurements performed using this instrument were the limbal and bulbar redness classification, lipid layer thickness (LLT), tear meniscus height (TMH), first and mean noninvasive tear break-up time (FNIBUT and MNIBUT, respectively) (objective and automatic), and Meibomian gland loss (MGL) percentage and grade (objective and automatic) [19].

Tear volume was measured by Schirmer’s I test (SIT) [20], and the invasive analysis of tear film stability was carried out through the fluorescein tear break-up time test (TFBUT) using cobalt blue illumination of a slit lamp (Topcon SL-6E, Japan). TFBUT was the average of the three measures of each eye [21].

### Examination procedure

After verification of compliance with the previously established criteria, subjects were included in the study.

This was followed by noninvasive examination of the tear film by OSA in the following order [19]: (I) limbal and bulbar redness. The degree of redness was assessed by comparing the image taken by the device with the Efron scale [22] (0 = normal, 1 = trace, 2 = mild, 3 = moderate, 4 = severe). Reddening values above grade 2 were considered abnormal. (II) Interferometric LLT. The interferometric patterns were classified into the 7 categories defined by Guillon [23] and their quantitative equivalence in LLT (from thinner to thicker: 0 < 15 nm – not present, 1 ~ 15 nm – open meshwork, 2 ~ 30 nm – close meshwork, 3 ~ 30/80 nm – wave, 4 ~ 80 nm – amorphous, 5 ~ 80/120 nm – color fringes, 6 ~ 120/160 nm – abnormal color). Categories below the wave pattern were considered abnormal and corresponded to reduced lipid layer thickness. (III) TMH. This test assessed the mucoaqueous layer of the tear. TMH was measured in millimeters along the lower lid margin at the center of the cornea by the magnification tool. TMH values ≤ 0.20 mm were considered abnormal [24]. (IV) Noninvasive tear break-up time. With this measurement, the tear film stability was evaluated. The device automatically provides the FNIBUT and MNIBUT in seconds (s). Values of MNIBUT < 10 s were considered abnormal and indicative of eye [25]. (V) Meibomian gland assessment. The percentage and degree of MGL were determined by noncontact infrared meibography and subsequent automatic analysis, or semiautomatic when this was not possible, on the lower and upper eyelids separately. The percentage of MGL was represented as the percentage of the area of missing glands in the region of the lower and upper tarsal plates and graded according to a 4-stage scale (Meiboscale) [26] (0 ~ 0%, 1 < 25%, 2 = 26% – 50%, 3 = 51% – 75%, 4 > 75%). An MGL value above 25% (grade 2) was considered abnormal.

Finally, invasive tear film analysis was performed using SIT and TFBUT. ST < 10 mm and TFBUT < 10 s were considered abnormal [25].

All examinations were performed at the same location, under the same temperature and humidity conditions, by an experienced examiner using the same instrumentation.

### Statistical analysis

Because there were no significant differences observed in measurements between the right and left eyes, data from one eye per patient were considered for further analysis, choosing the right eye or left eye by simple and computer-generated random numbers.

Data were analyzed using IBM SPSS® Statistics 26 for Windows version (IBM Corporation, Armonk, NY). The normality of the variables was checked using the Shapiro–Wilk test (*p* > 0.05). Quantitative variables that conformed to normal were described by the mean ± standard deviation (SD), and those that were not described, by the median (interquartile range). Comparisons between the two groups were analyzed using the independent *T* test (Mann–Whitney *U* test for nonparametric). The chi-square test was used for qualitative variables. The relationship between the variables considered was assessed using the correlation Pearson test (Spearman for nonparametric). For all comparisons, a *p* value < 0.05 was considered statistically significant.

The sample size was determined in the GRAMMO® calculator (Institut Municipal d’Investigació Mèdica, Barcelona, Spain, Version 8.0) based on the TMH as the main study variable. The common SD in the TMH is assumed to be 0.07 for the group with type 1 diabetes based on the results obtained by Han et al. [27]. Accepting an alpha risk of 0.05 and beta risk of 0.2 in a two-sided test, a minimum of 31 subjects are necessary in each group to recognize as statistically significant a difference greater than or equal to 0.05 mm in the TMH.

## Results

### Demographics and clinical characteristics

Eighty-eight subjects (36 males and 52 females) were enrolled in this study, including 44 patients with T1DM and 44 controls between 18 and 49 years old. All subjects were Spanish Caucasians. The demographics and clinical characteristics of the participants are shown in Table 1.

**Table 1:** Demographics and clinical characteristics of the participants in the study

Variable	T1DM group (*n* = 44)	Control group (*n* = 44)	*p* value
Age (years)	31.00 ± 10.49 (18 to 49)	30.09 ± 10.48 (18 to 49)	0.910
Male, n (%) Female, n (%)	16 (36) 28 (64)	20 (45) 24 (55)	0.386
CDVA (Log MAR)	− 0.086 ± 0.049 (0.04 to − 0.18)	− 0.094 ± 0.073 (0.06 to − 0.28)	0.499
HbA1c (%)	6.88 ± 0.64 (5.8 to 8.4)	4.99 ± 0.20 (4.6 to 5.4)	< 0.001^a^
Diabetes duration (years)	16.27 ± 9.03 (3 to 38)	0	–-
OSDI (score points)	12.50 (5.21, 22.50) (0 to 41.67)	8.33 (4.17, 18.75) (0 to 37.50)	0.637
SPEED (score points)	4.00 (1.50, 8.50) (0 to 16)	4.00 (2.00, 9.00) (0 to 12)	0.563

*CDVA* corrected distance visual acuity, *DMT1* type 1 diabetes mellitus, *IQR* interquartile range, *OSDI* Ocular Surface Disease Index, *SD* standard deviation, *SPEED* Standard Patient Evaluation of Eye Dryness

Values are presented as mean ± SD (range) or median (IQR) (range) in quantitative variables and as frequency (percentage) in qualitative variables

^a^Statistically significant differences through independent *T* test

The T1DM and control groups were similar with respect to age, sex, correct distance visual acuity, and subjective dry eye symptom questionnaires score points OSDI and SPEED; however, there was a statistically significant difference in the percentage of HbA1c between the two groups (*p* < 0.001). Scores on the OSDI and SPEED questionnaires were directly correlated (*ρ* = 0.697, *p* < 0.001).

### Noninvasive tear film test

Comparisons of the values obtained in the OSA tests between the two groups of participants, the statistical significance (*p* value), and subjects with abnormal values in each variable are summarized in Table 2.

**Table 2:** Comparison of the ocular surface analyzed parameters between the two groups of participants, and subjects (*n*, %) with abnormal values in each variable

Variables ^a^	T1DM group (*n* = 44)	Control group (*n* = 44)	*p* value	Subjects with abnormal values, *n* (%)	
				T1DM	Control
Limbal and bulbar redness (Efron scale)	2.00 (1.00,2.00)	1.00 (1.00,2.00)	0.010^b^	31 (70)	17 (39)
Tear meniscus height (mm)	0.20 (0.18, 0.23)	0.29 (0.24, 0.35)	˂0.00^b^	23 (52)	2 (8)
Lipid layer thickness (Guillon pattern)	1.00 (1.00, 2.00)	2.00 (2.0, 3.00)	˂0.001^b^	40 (91)	28 (64)
FNIBUT (seconds)	4.41 (3.87, 4.74)	5.14 (4.48, 5.56)	˂0.001^b^	-	-
MNIBUT (seconds)	8.65 (7.01, 9.60)	9.80 (9.15, 10.95)	˂0.001^b^	35 (86)	25 (57)
Upper eyelid MGL (percentage)	23.59 ± 10.43 22.50 (14.50, 31.50)	19.11 ± 12.57 19.00 (6.50, 30.00)	0.097	18 (41)	16 (36)
Lower eyelid MGL (percentage)	33.27 ± 10.20 36.00 (30.00, 41.50)	24.23 ± 10.89 22.00 (16.50, 30.50)	< 0.001^c^	40 (91)	16 (36)
Schirmer I test (mm/5 min)	5.00 (4.00, 6.50)	5.50 (6.00, 13.00)	˂0.001^b^	18 (41)	6 (14)
TFBUT (s)	13.00 (8.50, 25.00)	21.5 (15.50, 35.00)	0.001^b^	42 (96)	27 (61)

*T1DM* type 1 diabetes mellitus, *MGL* Meibomian glands loss, *FNIBUT* first noninvasive break-up time, *MNIBUT* mean noninvasive break-up time, *TFBUT* fluorescein break-up time test

^a^Values are presented as mean ± SD or median (IQR)

^b^Statistically significant differences by Mann–Whitney *U* test

^c^Statistically significant differences by independent *T* test

**Fig. 1:**
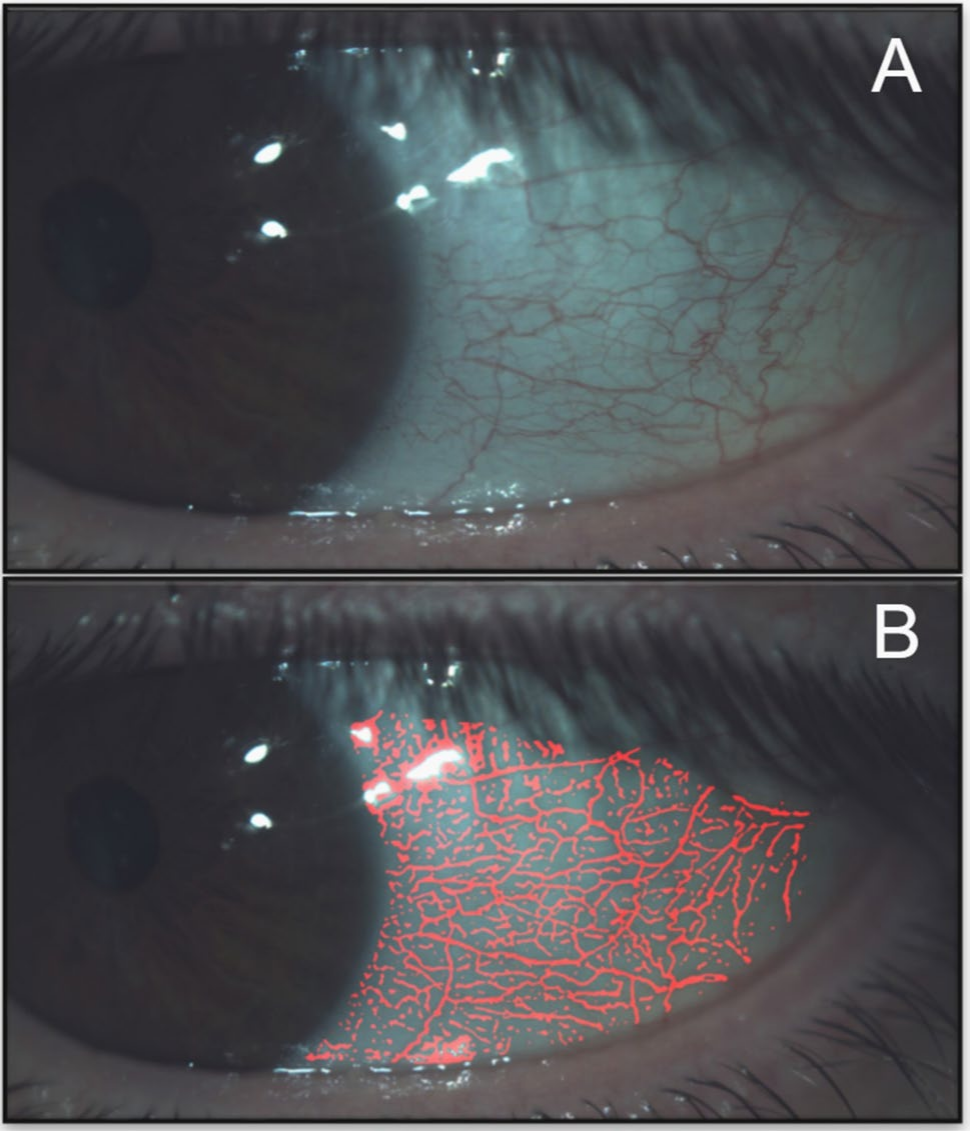
Bulbal and limbar redness classification as grade 3 according to the Efron scale [19]. **A** The left eye with temporal conjunctival blood vessels fluidity deactivated. **B** The same eye with conjunctival blood vessels fluidity activated

The T1DM group showed higher limbal and bulbar redness than the control group (*p* = 0.010). Fifty-four percent of the participants had conjunctival redness equal to or greater than grade 2 (mild) on the Efron scale [22], and 35% were in patients with T1DM compared to 19% without T1DM. An example of conjunctival redness examination is presented in Fig. 1.

**Fig. 2:**
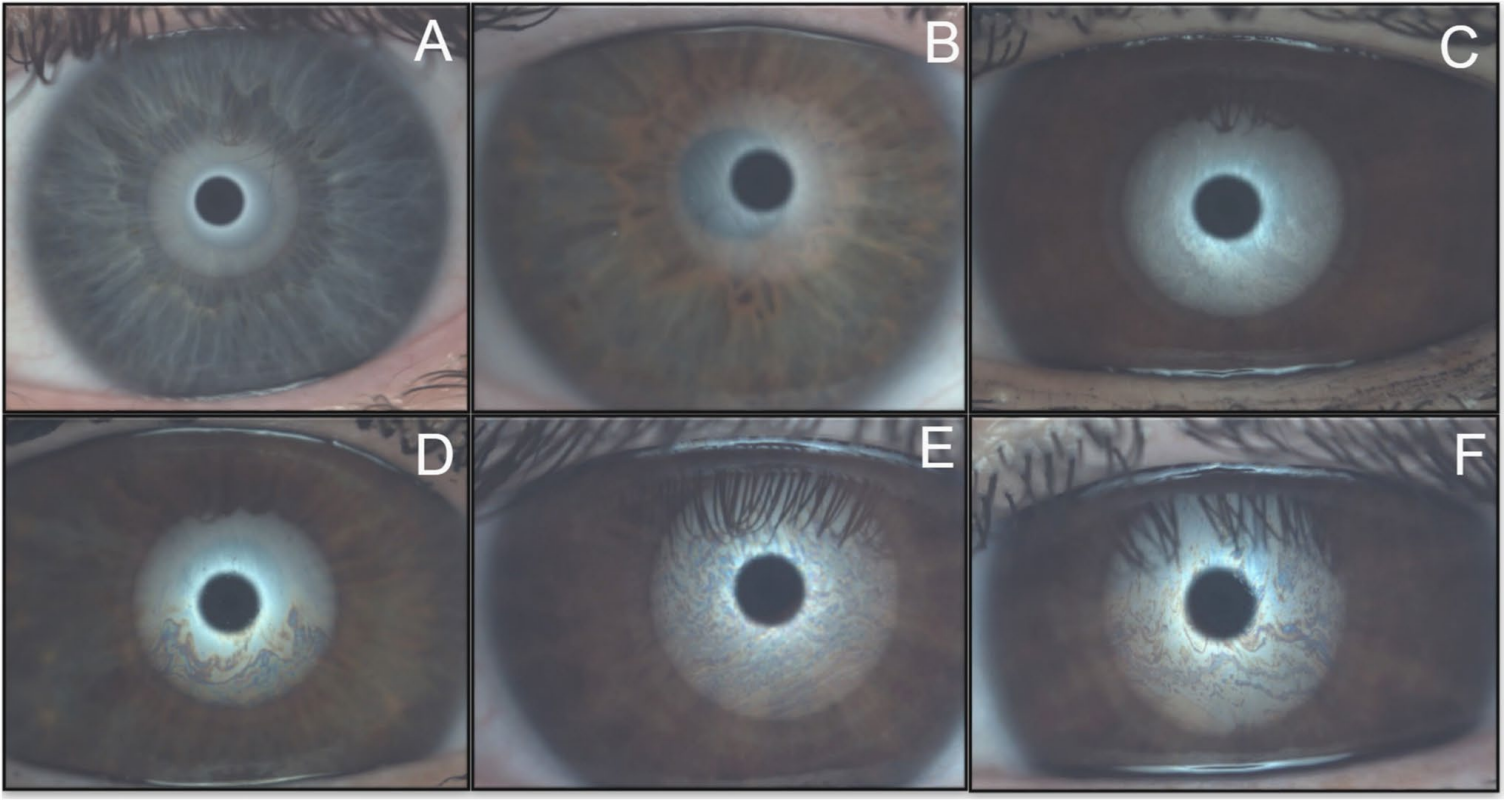
Examples of different interferometric lipid patterns found through the ocular surface analyzer (OSA). Classification was performed according to the categories defined by Guillon [19] and their quantitative equivalence in lipid layer thickness (LLT). **A** Not present (0 < 15 nm). **B** Open meshwork (~ 15 nm). **C** Close meshwork (~ 30 nm). **D** Wave (~ 30/80 nm). **E** Amorphous (~ 80 nm). **F** Color fringes (80/120 nm)

In the LLT assessment, lower lipid pattern values were obtained in the T1DM group than in the control group (*p* < 0.001). Seventy-seven percent of the participants (45% in the T1DM group and 32% in the control group) had a lipid pattern category below the wave pattern (~ 30/80 nm), thus reducing LLT. Examples of examinations of different lipid patterns found are presented in Fig. 2.

The TMH in the T1DM group was lower than that in the control group, with a statistically significant median difference of 0.09 mm (*p* < 0.001). Twenty-eight percent of the participants (26% with T1DM and 2% without T1DM) had a reduced meniscus height, indicating a decreased mucoaqueous layer.

In the assessment of tear film stability, patients with T1DM had a lower FNIBUT and MNIBUT than controls (*p* < 0.001). Sixty-six percent of participants (43% with T1DM and 28% without T1DM) presented tear film instability and therefore the presence of dry eye signs by showing an MNIBUT of less than 10 s [25].

### Meibomian gland assessment

**Fig. 3:**
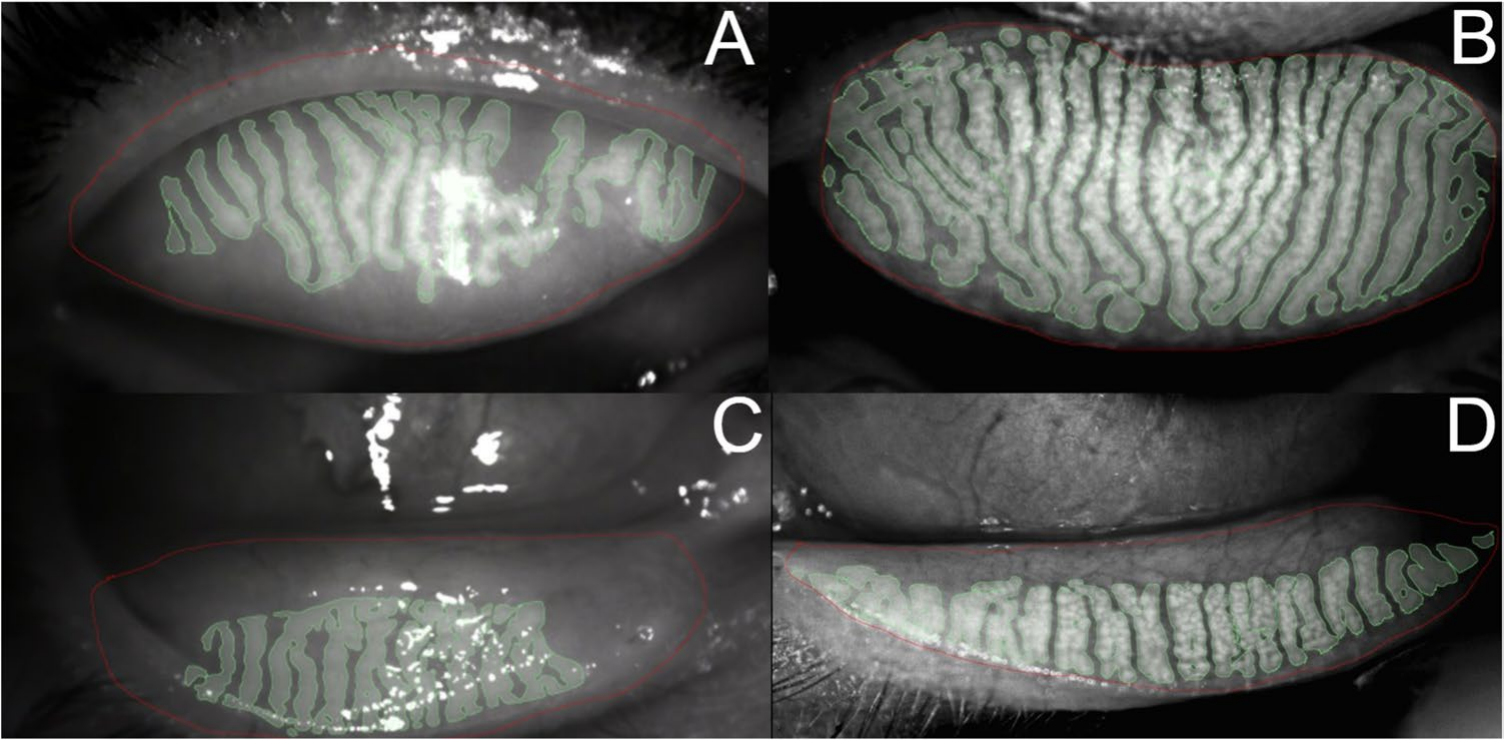
Meibomian gland assessment through the ocular surface analyzer (OSA). The percentage of Meibomian gland loss (MGL) was graded according to a 4-stage scale (Meiboscale) [23]. **A**, **C** The right eyes and **B**, **D** the left eyes. **A** The upper eyelid MGL = 31% (grade 2); **B** the upper eyelid MGL = 8% (grade 1); **C** the lower eyelid MGL = 58% (grade 3); **D** the lower eyelid MGL = 33% (grade 2)

A higher percentage of MGL was found in the T1DM group in the upper and lower eyelids, although the difference between the two groups was only statistically significant in the case of the lower eyelid (*p* < 0.001) (Table 2). Regarding the lower eyelid MGL percentage, in 64% of participants (46% with T1DM and 16 without T1DM), it was higher than 25% (grade 2). In contrast, the upper eyelid MGL percentage was higher than 25% (grade 2) in only 32% of the participants (20% with T1DM and 18% without T1DM). Some examples of upper and lower eyelid MGLs through OSA are shown in Fig. 3.

### Invasive tear film analysis

In the SIT, lower values were obtained in the T1DM group than in the control group (*p* = 0.001) (Table 2). Twenty-seven percent of the participants (20% with T1DM and 7% without T1DM) had a reduced tear volume according to this test. Regarding the TFBUT, the T1DM group also had a lower value than the control group (*p* < 0.001) (Table 2). Seventy-two percent of participants (48% with T1DM and 31% without T1DM) had values of less than 10 s, indicating tear film instability.

### Correlation analysis of HbA1c and diabetes duration with the tests

The percentage of HbA1c was directly correlated with limbal and bulbar redness (*ρ* = 0.226, *p* = 0.034) and lower eyelid MGD (*r* = 0.396, *p* < 0.001). In addition, HbA1c was inversely correlated with TMH (*ρ* =  − 0.584, *p* < 0.001), LLT (*ρ* =  − 0.361, *p* = 0.001), FNIBUT (*ρ* =  − 0.525, *p* < 0.001), MNIBUT (*ρ*= − 0.399, *p* < 0.001), SIT (*ρ* =  − 0.317, *p* = 0.003), and TFBUT (*ρ* =  − 0.566, *p* < 0.001).

In the T1DM group, an inverse correlation of diabetes duration with LLT (*ρ* =  − 0.400, *p* = 0.007) and a direct correlation of diabetes duration with upper eyelid MGD (*ρ* = 0.413, *p* = 0.005) were obtained.

## Discussion

In this study, tear film layers and MGs were assessed in adult patients with and without T1DM using a noninvasive ocular surface analyzer. A statistically significant impairment of the mucoaqueous and lipid layers, as well as a greater loss of lower eyelid MGs, was found in patients with T1DM. However, no significant differences were found in dry eye symptoms across the OSDI and SPEED questionnaires between the T1DM and control groups. This could be because some of the T1DM patients may have a lower corneal sensitivity caused by peripheral neuropathy [28]. Diabetic neuropathy is a serious complication of diabetes and the main cause of nerve damage [29]. In some cases, because of this, patients with T1DM may be asymptomatic even with severe damage to the ocular surface, indicating progression of peripheral neuropathy [30].

### Noninvasive tear film test

The tear film was quantified noninvasively, obtaining LLT and bulbar network measurements by optical interferometry, TMH, FNIBUT, and MNIBUT. In all of them, lower results were obtained in the T1DM group compared to the control group, except for bulbar redness, which was greater in the T1DM group, and the differences were also statistically significant in all of them. Several authors describe the differences in NIBUT between people with and without DM, although the methods of measurement are very different, and many measure it invasively (TFBUT) and in T2DM [31] or in children [10, 32], always describing lower values for patients with DM.

We cannot make an exhaustive comparison of the FNIBUT or the MNIBUT, two values of recent appearance with the use of noninvasive devices, which are usually compared with the reference values in subjective methods.

Few studies perform a complete noninvasive examination of the tear film, such as that of Zeng et al. [33], which found no differences between patients with DM and the control group in the TMH and NIBUTM measured with Keratograph, nor in the LLT, measured with the Tearscope, although in this case they are again T2DM; or Garzon P et al. [34] that quantifies NIBUT, LLT with interferometry, and TMH with OCT in T2DM, also obtaining lower values, but without statistically significant differences. Additionally, Han et al. [27] used a dry eye analyzer similar to the one used in the present study, describing worse results in the analyzed group, although they were again T2DM patients; similarly, Nadeem et al. [28] described a lower TMH and no relationship between this loss and the duration of the disease and who also suffered from diabetic retinopathy.

Only Misra et al. [35] noninvasively analyzed NIBUT and LLT in patients with T1DM, obtaining lower values. Only the former was significant, but these patients were diagnosed with corneal neuropathy.

### Meibomian gland assessment

Patients with T1DM showed a higher percentage of MGL than patients without T1DM, with the highest value found in the lower eyelid of patients with T1DM, grade 2 according to the Meiboscale [26]. Eom et al. [36] reported a statistically significant higher lower eyelid and upper eyelid MGL in patients with obstructive MGD, but no studies have been found in people with T1DM. Because of MGD, a reduction in the lipid layer was also found in this study [37]. The relationship of MGD with T2DM has been studied by several authors [9, 20, 33, 34, 38–40], mostly indicating a higher MGD in these patients; however, studies in the population with T1DM are more limited, and this is the first one in an adult population. There are recent publications in children and adolescents with T1DM [41, 42]. In the study by Koca et al. [41], children with T1DM had a higher percentage of GML than controls, although this did not correlate with HbA1c or duration of diabetes; however, the study by Gunay et al. [42] reported certain altered ocular surface parameters but no differences with controls with respect to MGL.

### Invasive tear film tests

Participants with T1DM had a reduced tear volume compared to controls in the SIT. These results are consistent with other research in adults with T1DM [43, 44], children and adolescents [42, 45], and subjects with T2DM [33, 46–49] but disagree with Ferdousi et al. [50]. Shujaat et al. [45] suggested that diabetes may cause damage to the microvasculature and innervation of the main lacrimal gland, affecting tearing.

Regarding TFBUT, patients with T1DM had lower TFBUT values than controls, corroborating the results of other authors [45, 50] and supporting the results obtained in the NIBUT, although there are also some authors who disagree with this result.

It is logical to suppose that if SIT values are lower in patients suffering from DM, as has been described by some authors, and with which we agree, and the MG are more affected, we would find lower values of LLC and TMH. This would be related to the fact that the mean value of bulbar reddening is higher in T1DM, also considering that the mucoaqueous layer of the tear is reduced in this group.

### Correlation analysis of HbA1c and diabetes duration with the tests

The percentage of HbA1c correlated significantly with all tests performed except the upper eyelid MGL, indicating a relationship between high HbA1c values and deterioration of the tear film layers and the lower eyelid MGL. Tavakoli et al. [51] indicated a significant inverse correlation between HbA1c and corneal nerve fiber density, which would indicate the protective factor of good glycemic control against corneal complications.

A statistically significant moderate relationship of diabetes duration with lower LLT (*p* = 0.007) and higher upper eyelid MGL (*p* = 0.005) was also found, showing a direct relationship with the presence of evaporative-type dry eye. Other studies have also indicated that tear film instability increases with diabetes duration, finding a relationship with TFBUT in patients with T1DM [10, 45].

### Limitations

Given the cross-sectional design, this study only reports a relationship between the factors and the results obtained, but we cannot draw conclusions about the cause. Future longitudinal studies are needed to confirm these results. Moreover, an examiner masked to the type of patient would reduce the risk of bias. In addition, a larger sample size would be recommended to be able to group patients according to HbA1c percentage, duration of diabetes, age, or sex.

## Conclusion

Patients with T1DM without signs of retinopathy showed involvement of the mucoaqueous and lipid layers of the tear film, as well as a higher percentage of MGL, using a noninvasive analyzer. DED in people with T1DM cannot be ruled out by anamnesis and subjective symptom questionnaires alone; therefore, these patients should undergo regular anterior pole examinations. Noninvasive instruments, such as the OSA, are useful to make a rapid diagnosis of dry eye signs, causing less discomfort to patients than invasive methods.

## Data Availability

The data showed in this research are available on request from the corresponding author. The data are non-publicly available due to their containing information that could compromise the privacy of research participants.
